# Tetrahydrofolate Alleviates the Inhibitory Effect of Oxidative Stress on Neural Stem Cell Proliferation through PTEN/Akt/mTOR Pathway

**DOI:** 10.1155/2022/9021474

**Published:** 2022-02-27

**Authors:** Xuyang Zhang, Zhi Liu, Wenqin Yang, Fengchun Zhao, Chao Zhang, Hui Feng, Tengyuan Zhou, Jun Zhong, Yongjie Zou, Hua Feng, Hongfei Ge, Rong Hu

**Affiliations:** ^1^Department of Neurosurgery and Key Laboratory of Neurotrauma, Southwest Hospital, Third Military Medical University (Army Medical University), 400038 Chongqing, China; ^2^Department of Neurosurgery, The 908th Hospital of Chinese People's Liberation Army Joint Logistic Support Force, 330002 Nanchang, Jiangxi, China

## Abstract

Neural stem cell (NSC) proliferation is the initial step for NSC participating in neurorehabilitation after central nervous system (CNS) injury. During this process, oxidative stress is always involved in restricting the regenerative ability of NSC. Tetrahydrofolate (THF) is susceptible to oxidative stress and exhibits a high antioxidant activity. While its effect on NSC proliferation under oxidative stress condition remains obscure. Here, NSC were isolated from embryonic mice and identified using immunofluorescent staining. Meanwhile, the results showed that THF (5 *μ*M and 10 *μ*M) attenuated oxidative stress induced by 50 *μ*M hydrogen peroxide (H_2_O_2_) in NSC using mitochondrial hydroxyl radical detection and Western blotting assays. Afterward, administration of THF markedly alleviated the inhibitory effect of oxidative stress on NSC proliferation, which was evidenced by Cell Counting Kit-8 (CCK8), neurosphere formation, and immunofluorescence of Ki67 assays. Thereafter, the results revealed that PTEN/Akt/mTOR signaling pathway played a pivotal role in counteracting oxidative stress to rescue the inhibitory effect of oxidative stress on NSC proliferation using Western blotting assays and gene knockdown techniques. Collectively, these results demonstrate that THF mitigates the inhibitory effect of oxidative stress on NSC proliferation via PTEN/Akt/mTOR signaling pathway, which provides evidence for administrating THF to potentiate the neuro-reparative capacity of NSC in the treatment of CNS diseases with the presence of oxidative stress.

## 1. Introduction

Neural stem cell (NSC), residing in the dentate gyrus (DG) of hippocampus and subventricular zone (SVZ) of the lateral ventricle, are the main regenerative neural cell subtype in the central nervous system (CNS) [1–4]. Under physiological condition, NSC dynamically keep in a delicate balance among self-renewal, differentiation, and quiescence [5]. While under pathological situation, NSC hold the ability of proliferating *in situ*, migrating toward the injured regions, differentiating into three main neural subpopulations (neurons, astrocytes, and oligodendrocytes), and integrating into the damaged neurovascular network to accelerate functional recovery after CNS injury [6–9]. NSC proliferation is the initial step for NSC taking part in neurorehabilitation after CNS injury. Several factors have been identified to be associated with NSC proliferation.

The level of reactive oxygen species (ROS) is one of the factors mediating NSC proliferation. At steady state, low basal dynamic ROS levels maintain NSC in a sophisticated balance among self-renewal, differentiation, and quiescence [5]. Previous studies have indicated that a slightly higher ROS level boosts NSC proliferation isolated from SVZ [5, 10–12], whereas excessive level of ROS, characterized as ROS surplus, always results in oxidative stress [13]. When oxidative stress persists, NSC are prone to suffer from maintaining in quiescence, that is, NSC lose proliferation potential or lack of self-renewal capacity [13, 14]. Considering that oxidative stress is often introduced in subjects with the ictus and progression of CNS injury, such as traumatic brain injury (TBI) [15, 16], cerebral stroke [17–19], and neurodegenerative diseases [20–22], exploring interventions to alleviate oxidative stress might be an effective therapeutic strategy to strengthen the rehabilitative ability of NSC after CNS injury.

Tetrahydrofolate (THF), the active form of folate (vitamin B9), is an essential cofactor of one-carbon (1C) cycle and involved in the biosynthesis of thymidylate, purines, and glycine [23, 24]. Previous researches have delineated that THF is susceptible to oxidative stress and exhibits a high antioxidant activity when excessive level of ROS cumulation is available [25, 26], implying that THF might be a candidate for ameliorating surplus ROS to enhance rehabilitative capacity of NSC. Meanwhile, previous study has demonstrated that the THF transporter of SLC25A32 is highly expressed in varying tumors, and obviously facilitated tumor cell growth to reduce patients' survival, whereas downregulation of SLC25A32 inhibits tumor cell propagation [27]. Moreover, THF administration is an effective intervention in promoting proliferation of dihydrofolate reductase- (DHFR-) deficient Chinese hamster ovary (CHO) DG44 cells [24], while the effect of THF on NSC proliferation and the underlying mechanism remains largely unknown.

Phosphatase and tensin homologue (PTEN) usually serves as a negative modulator in maintaining NSC proliferation [28]. Recently, investigation shows that PTEN/protein kinase B (Akt) signaling pathway exerts neuroprotective effect on spiral ganglion neuron after oxidative stress exposure in a manner of negatively regulating Akt [29]. Additionally, previous studies have demonstrated that Akt/mammalian target of rapamycin (mTOR) signaling pathway is an essential regulator for NSC proliferation [30–33] and participates in manipulating redox homeostasis in macrophage and glioma cells [34–36]. Thereafter, a hypothesis that THF administration might mitigate the inhibitory effect of oxidative stress on NSC proliferation due to PTEN/Akt/mTOR pathway is issued in the present study.

To test this hypothesis, NSC were isolated from embryonic mice and identified using phase-contrast observation and immunofluorescence. Next, the hydrogen peroxide (H_2_O_2_) was used to introduce oxidative stress, and the level of mitochondrial ROS (MitoROS) was determined using mitochondrial hydroxyl radical detection assays. Afterward, the effect of THF on NSC proliferation under oxidative stress condition was determined by Cell Counting Kit-8 (CCK8), neurosphere formation, and immunofluorescence of Ki67 assays. Subsequently, the underlying mechanism was explored using Western blotting assays and gene knockdown techniques. The aim of the present study is to provide a rationale for administrating THF to potentiate the restorative ability of NSC in the treatment of CNS diseases with the presence of oxidative stress.

## 2. Materials and Methods

### 2.1. Animals

Embryonic C57BL/6 mice were purchased from the Third Military Medical University (Army Medical University). All animal experiments were performed in accordance with China's animal welfare legislation for protection of animals used for scientific research and were approved by the local authorities of the Third Military Medical University (Army Medical University) for the laboratory use of animals (approval no. SCXK-20170002).

### 2.2. NSC Culture and Treatment

Primary NSC were isolated and cultured as previously described [9, 37]. Briefly, the cerebral cortex was dissected and washed twice with DMEM (Gibco, Grand Island, NY). After peeling off the meninges, tissues were incubated in 0.25% trypsin-EDTA (Gibco, Grand Island, NY) at 37°C for 30 minutes, then washed twice in DMEM after being incubating in 10% fetal bovine serum (FBS, Gibco, Grand Island, NY) for 2 times. Finally, tissues were suspended in stem cell culture medium as follows: DMEM/F12 medium supplemented with 2% B27 (Gibco, Grand Island, NY), 20 ng/ml EGF (Peprotech, Rocky Hill, NJ) and 20 ng/ml FGF-2 (Peprotech, Rocky Hill, NJ). Afterward, cell suspension was passed through a 70 *μ*m Nylon cell strainer (BD Falcon, San Jose, CA). Subsequently, NSC suspension was incubated at 37°C under 5% CO_2_ humidified condition. The procedures for NSC passage were performed according to standard methods as described in our previous researches [9, 37]. In order to make NSC grow adherently, 10 *μ*g/ml poly-L-ornithine (Sigma-Aldrich, St. Louis, MO) was used to precoat cell culture plates. NSC were allowed to grow at a density of 1 × 10^5^ for 24 hours at 37°C prior to implementing the experiments. NSC were pretreated with THF for 30 minutes and then stimulated with 50 *μ*M H_2_O_2_. THF (Sigma-Aldrich, St. Louis, MO) and H_2_O_2_ (Lircon, Shandong, China) were dissolved in PBS, and diluted to specific concentration by NSC culture medium. Meanwhile, the equal volume of PBS was added as control or scramble group.

### 2.3. NSC Differentiation

NSC were seeded on confocal culture dishes precoated with 10 *μ*g/ml poly-L-ornithine (PLO). Then, cells were incubated in DMEM/F12 containing 1% glutamate (Gibco, Grand Island, NY) and 2% B27 (Gibco, Grand Island, NY) for 7 days.

### 2.4. Cell Viability Assays

Cell Counting Kit-8 (CCK8) assays were performed to assess the viability of NSC. Approximately 2 × 10^4^ single NSC in 200 *μ*l culture medium were seeded in 96-well plates for 24 hours, then treated with different concentrations of THF. Subsequently, 50 *μ*M H_2_O_2_ was added after 30 minutes. Thereafter, 20 *μ*l CCK-8 working solution (Boster, Wuhan, China) was added into each well and incubated at 37°C for 2 hours. The absorbance value was determined with a microplate reader at 450 nm.

### 2.5. Growth Curve Analysis

NSC were seeded in 12-well plates at a density of 1 × 10^5^ single cell per well. Then, NSC in each group were treated with H_2_O_2_ or THF for 0, 24, 48, and 72 hours. Thereafter, cells were counted with a haemocytometer after being suspended in culture medium.

### 2.6. Mitochondrial Hydroxyl Radical Detection

The test was performed according to the manufacturers' instructions of Cell Meter™ Mitochondrial Hydroxyl Radical Detection Kit (AAT Bioquest, USA). Briefly, NSC were seeded onto 10 *μ*g/ml PLO-coated confocal culture dishes and incubated for 24 hours. Then, they were incubated in H_2_O_2_ or THF for another 24 hours. Afterward, the culture medium was removed, and 1 ml MitoROS™ OH580 working solution was added to each confocal dish at 37°C for 60 minutes. Thereafter, cells were washed 2-3 times with Hank's Balanced Salt Solution (HBSS), and Hoechst dye solution (Beyotime, China) was diluted to each well for 15 minutes. The fluorescence density was determined using a Confocal Laser Microscopy (Carl Zeiss, LSM880, Weimar, Germany) at Ex/Em = 540/590 nm with bottom read mode and examined by Zen 2011 software (Carl Zeiss, Weimar, Germany).

### 2.7. Neurosphere Formation Assay

Approximate amount of 1000 single NSC were cultured in uncoated 12-well plate for 24 hours. NSC in each group were suffered from different treatments. Then, the number and average diameter were observed using a phase-contrast microscopy (IX71, Olympus, Tokyo) at 24 hours and 48 hours.

### 2.8. PTEN Knockdown

The procedures for PTEN knockdown were performed according to the manufacturer's instruction. PTEN shRNA lentivirus were purchased from Beijing Syngentech Co., Ltd. (Beijing, China). NSC were infected with PTEN-1 shRNA lentivirus at 70 multiplicity of infection (MOI) or PTEN-2 shRNA lentivirus at 100 MOI. Then, the PTEN knockdown efficiency was assessed using Western blotting assays at 4 days. The following target sequences were used in the present experiments: PTEN-1: CGACTTAGACTTGACCTATAT; PTEN-2: TTGTGGCAACAGATAAGTTTG.

### 2.9. Immunofluorescence

NSC were cultured in separated confocal culture dishes with different treatments. Afterward, NSC adhered to confocal culture dishes were fixed with 4% paraformaldehyde (PFA) for 30 minutes at room temperature and followed by 0.5% Triton X-100 in PBS for 30 minutes, then blocked with 5% BSA for 2 hours after washing with PBS for three times. After that, the cells were incubated with mouse anti-Nestin (1 : 200, Abcam, Cambridge, UK), rabbit anti-Sox2 (1 : 100, CST, USA), rabbit anti-Ki67 (1 : 400, CST, USA), rabbit anti-GFAP (1 : 800, Abcam, Cambridge, UK), rabbit anti-MAP2 (1 : 400, CST, USA), or rabbit anti-Olig2 (1 : 100, Proteintech Group, Inc., Chicago, IL, USA) antibodies overnight at 4°C. After washing with PBS for three times, the corresponding fluorescence secondary antibodies were incubated for 2 hours at room temperature, then cell nuclei were counterstained with 4′,6-diamidino-2-phenylindole (DAPI) for 20 minutes at room temperature. Finally, samples were visualized with a confocal microscope (Carl Zeiss, LSM780, Weimar, Germany) and examined by a Zen 2011 software (Carl Zeiss, Weimar, Germany).

### 2.10. Western Blotting

Samples in different groups were lysed by radio immunoprecipitation assay lysis buffer (RIPA; Beyotime, China) containing protease and phosphatase inhibitor cocktail (Roche, Indianapolis, IN, USA). Proteins of the same quantity (30 *μ*g/lane) in each group were separated by 10% or 15% SDS-PAGE and transferred to polyvinylidene difluoride (PVDF) membranes (Millipore, Temecula, CA, USA). Then, specimens were blocked by 10% skimmed milk in TBST at room temperature for 2 hours. Afterward, they were incubated with rabbit anti-SOD1 (1 : 1000, CST, USA), rabbit anti-SOD2 (1 : 1000, CST, USA), rabbit anti-PTEN (1 : 1000, CST, USA), rabbit anti-Phospho-Akt (1 : 1000, CST, USA), rabbit anti-Akt (1 : 1000, CST, USA), rabbit anti-Phospho-mTOR (1 : 1000, CST, USA), rabbit anti-mTOR (1 : 1000, CST, USA), mouse anti-GAPDH (1 : 1000, Santa Cruz Biotechnology, CA, USA), or mouse anti-*β*-actin (1 : 1000, Santa Cruz Biotechnology, CA, USA) antibodies overnight at 4°C. Secondary anti-mouse and anti-rabbit horseradish peroxidase- (HRP-) conjugated antibodies (1 : 10000, Boster, Wuhan, China) were used to conjoin the primary antibodies at room temperature for 2 hours. Finally, blots were visualized by electrochemiluminescence (ECL) substrate (ThermoFisher, USA). Signals were detected by Image Lab™ software (Bio-Rad, California, USA) and analyzed by Image Lab™ software (Bio-Rad, California, USA).

### 2.11. Statistical Analysis

All data were expressed as the mean ± S.D. and analyzed using SPSS 23.0 (SPSS Inc., Chicago, IL). Multiple comparisons were performed by one-way analysis of variance (ANOVA) and followed by Turkey's post hoc test if the data were normal distribution using a Shapiro–Wilk normality test. A *p* < 0.05 was considered to be significantly different.

## 3. Results

### 3.1. Primary NSC Isolation and Characteristics

**Figure 1:**
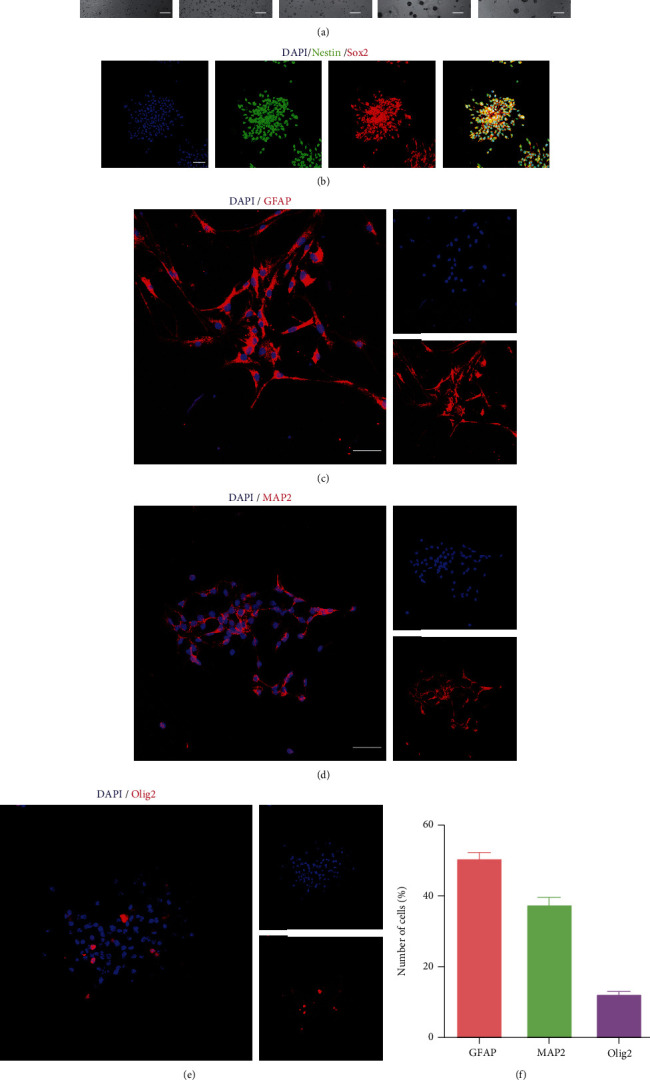
NSC culture and characteristics. (a) Morphological changes of cultured NSC under phase-contrast microscope from 6 to 48 hours in floating condition. Scale bar: 50 *μ*m. (b) Immunostaining images represented that the majority of cultured cells were positive for Nestin and/or Sox2. Scale bar: 20 *μ*m. (c) Immunostaining images showed that cultured cells could differentiate into GFAP^+^ cells after 7 days in differentiation medium. Scale bars: 10 *μ*m. (d) Immunostaining pictures indicated that cultured cells held the potential of differentiation into MAP2^+^ cells after 7 days in differentiation medium. Scale bars: 10 *μ*m. (e) Immunostaining images presented cultured cells possessed the capacity of transformation into Olig2^+^ cells after 7 days in differentiation medium. Scale bars: 10 *μ*m. (f) Histogram summarizing the percentage of cultured cell differentiation into GFAP^+^, MAP2^+^, and Olig2^+^ cells, respectively.

NSC were isolated from cerebral cortex of embryonic mice and cultured in floating condition. As shown in Figure 1(a), NSC were gradually formed from single cell to neurospheres after 48 hours. Then, most of the cultured cells exhibited Nestin^+^ and Sox2^+^, which are two main NSC markers [38], using immunofluorescence (Figure 1(b)). Thereafter, the differentiating potency of the NSC was determined by immunofluorescence after cultured in differentiation medium for 7 days. The immunostaining images showed that the cultured NSC held the potency of differentiating into about 50.2% of GFAP^+^ astrocytes, 36.8% of MAP2^+^ neurons, and 13% of Olig2^+^ oligodendrocytes (Figures 1(c)–1(f)), indicating that the cultured NSC presented stemness and differentiating potential into astrocytes, neurons, and oligodendrocytes.

### 3.2. THF Attenuated Oxidative Stress Induced by H_2_O_2_ in NSC

**Figure 2:**
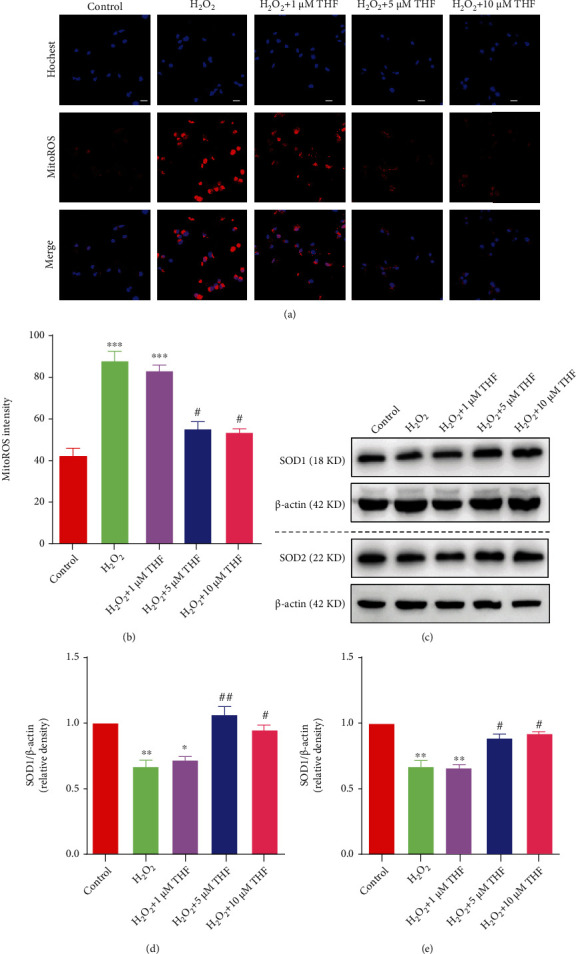
THF attenuated oxidative stress derived from H_2_O_2_ in NSC. (a) Mitochondrial ROS level in different groups. Scale bar: 10 *μ*m. (b) Bar graph illustrating the optic intensity from (a). *N* = 6, ^∗∗∗^*p* < 0.01 vs. control group; ^#^*p* < 0.05 vs. H_2_O_2_ group. (c) Bands demonstrating the expression of SOD1 and SOD2 in each group. *β*-Actin was used as an internal control. (d) Semiquantitative analysis of SOD1 from (c). *N* = 3, ^∗^*p* < 0.05, ^∗∗^*p* < 0.01 vs. control group; ^#^*p* < 0.05, ^##^*p* < 0.01 vs. H_2_O_2_ group. (e) Semiquantitation of SOD2 from (c). *N* = 3, ^∗^*p* < 0.05, ^∗∗^*p* < 0.01 vs. control group; ^#^*p* < 0.05, ^##^*p* < 0.01 vs. H_2_O_2_ group.

To determine the effect of THF on NSC proliferation under oxidative stress condition, we firstly evaluated the effect of THF on the production of reactive oxygen species (ROS) in NSC. To introduce oxidative stress in NSC, 50 *μ*M hydrogen peroxide (H_2_O_2_) was used. Meanwhile, three concentrations of THF (1 *μ*M, 5 *μ*M, and 10 *μ*M) were used to evaluate the optimal working concentration for relieving oxidative stress. The results showed that, compared with control group, the fluorescence intensity of MitoROS in H_2_O_2_ group was obviously increased (Figures 2(a) and 2(b)). While this effect was evidently abrogated after treatment with 5 *μ*M and 10 *μ*M THF, no dramatic difference was observed in 1 *μ*M THF (Figures 2(a) and 2(b)). Furthermore, the expression level of SOD1 and SOD2 in H_2_O_2_ and H_2_O_2_+1 *μ*M THF groups was significantly decreased, whereas this reduced expression level of SOD1 and SOD2 was partially reversed after application of 5 *μ*M and 10 *μ*M THF (Figures 2(c)–2(e)). These results indicated that the administration of 5 *μ*M and 10 *μ*M THF markedly attenuated oxidative stress, in comparison with that in control group. Therefore, 5 *μ*M THF was used in subsequent experiments in the present study.

### 3.3. THF Alleviated the Inhibitory Effect of Oxidative Stress on NSC Proliferation

**Figure 3:**
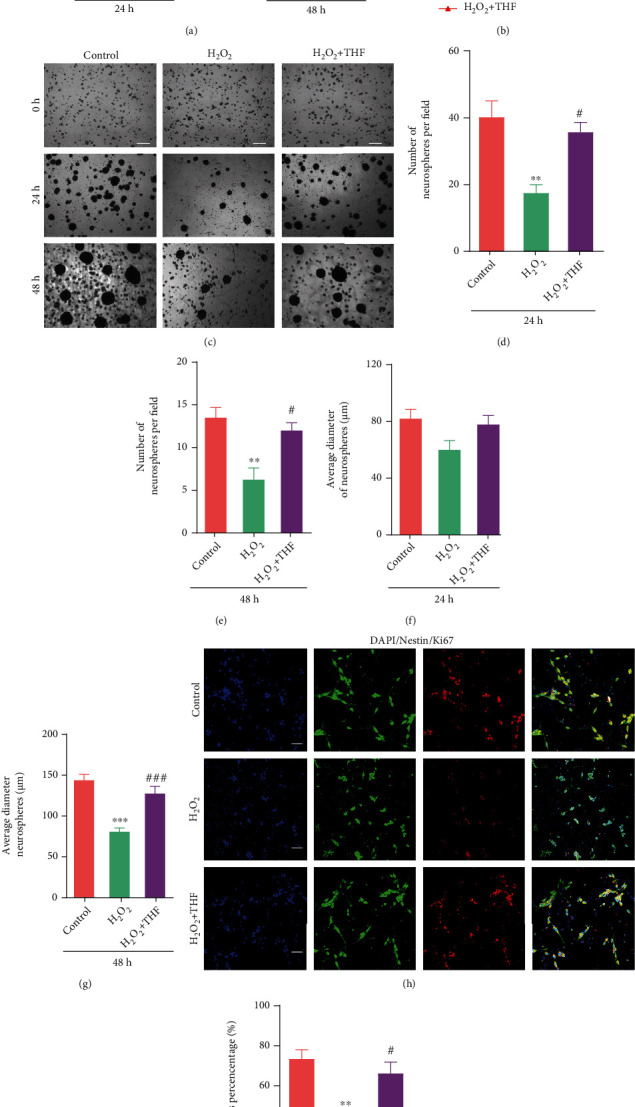
THF reversed the inhibitory effect of oxidative stress on NSC proliferation. (a) The absorbance value in each group at 24 h and 48 h using CCK-8 assays. *N* = 6, ^∗∗^*p* < 0.01 vs. control group; ^#^*p* < 0.05 vs. H_2_O_2_ group. (b) Growth curve analysis in different groups. *N* = 4, ^∗∗^*p* < 0.01, ^∗∗∗^*p* < 0.001 vs. control group; ^#^*p* < 0.05, ^##^*p* < 0.01 vs. H_2_O_2_ group. (c) The phase-contrasted images depicting NSC condition in each group at different timepoints. Scale bars: 50 *μ*m. (d) Bar graph summarizing the number of neurospheres per field among different groups at 24 h. *N* = 5, ^∗∗^*p* < 0.01 vs. control group; ^#^*p* < 0.05 vs. H_2_O_2_ group. (e) Bar chart representing the number of neurospheres per field among different groups at 48 h. *N* = 5, ^∗∗^*p* < 0.01 vs. control group; ^#^*p* < 0.05 vs. H_2_O_2_ group. (f) Histogram illustrating the average diameter of neurospheres in each group at 24 h. (g) Qualification of the average diameter of neurospheres in each group at 48 h. *N* = 5, ^∗∗∗^*p* < 0.001 vs. control group; ^###^*p* < 0.001 vs. H_2_O_2_ group. (h) The immunostaining of Nestin and Ki67 in various groups. Scale bars: 10 *μ*m. (i) The statistical analysis of Nestin^+^Ki67^+^ from (h). *N* = 6, ^∗∗^*p* < 0.01 vs. control group; ^#^*p* < 0.05 vs. H_2_O_2_ group.

To quantitatively assess the effect of THF on NSC proliferation under oxidative stress condition, CCK8 assays were firstly performed to elucidate cell viability in varying groups. The results showed that cell viability was significantly decreased when NSC were exposed to H_2_O_2_ for 24 hours and 48 hours (Figure 3(a)). Interestingly, cell viability was significantly increased when treated with 5 *μ*M THF compared with H_2_O_2_ group, but there was no significant difference between H_2_O_2_ + THF group and control group (Figure 3(a)). Then, the growth curve analysis indicated that oxidative stress derived from H_2_O_2_ inhibited NSC proliferation, while THF partially restored the ability of NSC proliferation (Figure 3(b)). Furthermore, neurosphere formation assays demonstrated that oxidative stress reduced the number of neurospheres at 24 hours and 48 hours in H_2_O_2_ group (Figures 3(c)–3(e)). Meanwhile, the average diameter of neurospheres was obviously diminished in H_2_O_2_ group at 48 hours, while THF could alleviate this effect compared with control group (Figures 3(f) and 3(g)). In addition, the immunostaining of Nestin and Ki67 was applied to validate the effect of THF on NSC proliferation triggering by oxidative stress. The immunostaining images represented that the percentage of Nestin and Ki67 double-positive cells in H_2_O_2_ group was surely lower than that in control group, and the proportion of Nestin and Ki67 double-positive cells was significantly higher in H_2_O_2_ + THF group than that in H_2_O_2_ group (Figures 3(h) and 3(i)). Together, these results revealed that the administration of THF alleviated the inhibitory effect of oxidative stress on NSC proliferation.

### 3.4. THF Alleviated the Inhibitory Effect of Oxidative Stress on NSC Proliferation through PTEN/Akt/mTOR Signaling Pathway

**Figure 4:**
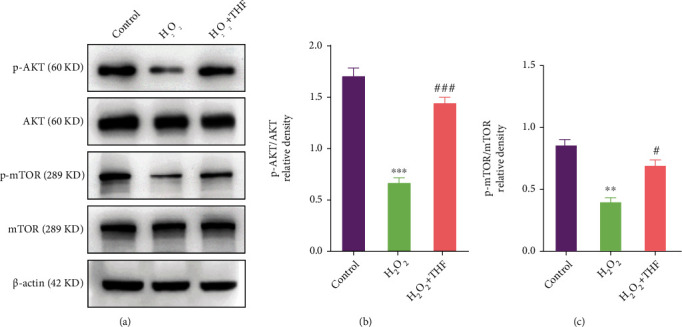
THF alleviated the inhibitory effect of oxidative stress on NSC proliferation through Akt/mTOR signaling pathway. (a) Bands depicted the expression of p-Akt, Akt, p-mTOR, and mTOR in different groups. (b) Semiquantitative analysis of p-Akt/Akt in control, H_2_O_2_, and H_2_O_2_ + THF groups from (a). *N* = 3, ^∗∗^*p* < 0.01 vs. control group; ^#^*p* < 0.05, ^##^*p* < 0.01 vs. H_2_O_2_ group. (c) Semiquantification of the expression of p-mTOR/mTOR from (a). *N* = 3, ^∗∗^*p* < 0.01 vs. control group; ^#^*p* < 0.05, ^##^*p* < 0.01 vs. H_2_O_2_ group.

Given that Akt/mTOR signaling pathway is a pivotal mediator for NSC expansion [30–33] and plays an important role in counteracting oxidative stress in macrophage and glioma cells [34–36], we then posited that Akt/mTOR signaling pathway was involved in regulating NSC proliferation resulting from oxidative stress. Herein, the expression of p-Akt and p-mTOR was determined using Western blotting. The bands depicted that the expression of p-Akt and p-mTOR was clearly downregulated in H_2_O_2_ group (Figures 4(a)–4(c)), while the reduced expression of p-Akt and p-mTOR was dramatically increased with the administration of 5 *μ*M THF (Figures 4(a)–4(c)).

**Figure 5:**
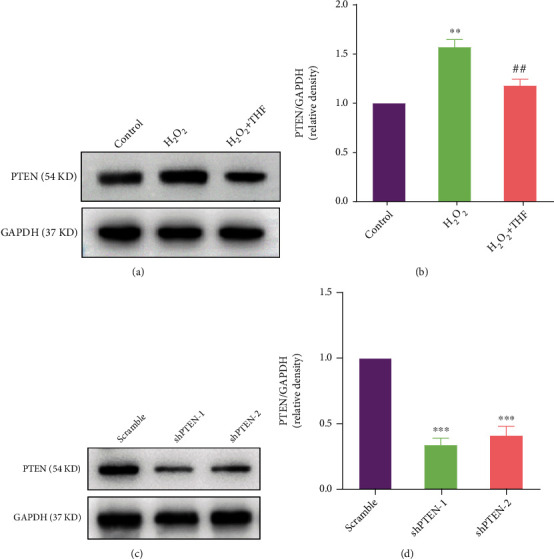
PTEN participated in THF partially abrogating the inhibitory effect induced by oxidative stress on NSC proliferation. (a) Immunoblot bands showing the expression of PTEN in control, H_2_O_2_, and H_2_O_2_ + THF groups. (b) Semiquantitative analysis of PTEN from (a). *N* = 3, ^∗∗^*p* < 0.01 vs. control group; ^##^*p* < 0.01 vs. H_2_O_2_ group. (c) Bands depicting the efficiency of PTEN KD in NSC. (d) Semiquantification from (c). *N* = 3, ^∗∗∗^*p* < 0.001 vs. scramble group.

Considering that PTEN is the upstream effector of Akt/mTOR signaling pathway and usually exerts neuroprotective effect on spiral ganglion neuron in response to oxidative stress [29], the expression of PTEN was assessed using Western blotting. Compared with control group, the expression of PTEN was significantly increased in H_2_O_2_ group (Figures 5(a) and 5(b)). To the contrary, the expression of PTEN in H_2_O_2_ + THF group was surely decreased (Figures 5(a) and 5(b)). Combined with the signaling pathway of Akt/mTOR aforementioned above, the results preliminarily certified the hypothesis that PTEN/Akt/mTOR signaling pathway played an evident role in the process that THF administration mitigated the inhibitory effect of oxidative stress on NSC proliferation.

**Figure 6:**
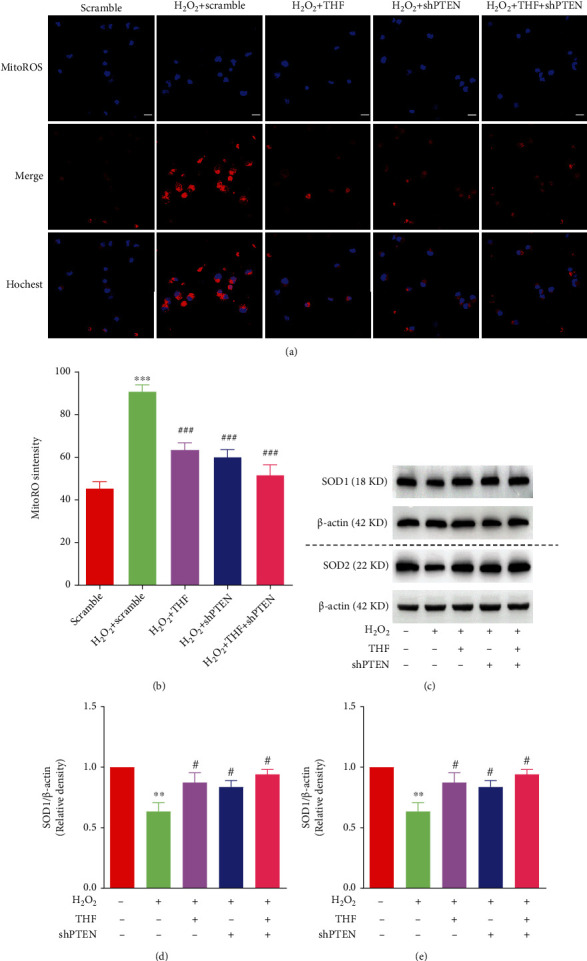
PTEN KD played the same role as THF in reducing oxidative stress in NSC. (a) Mitochondrial ROS level in different groups. Scale bar: 10 *μ*m. (b) Bar graph illustrating the optic intensity from (a). *N* = 4, ^∗∗∗^*p* < 0.001 vs. scramble group; ^###^*p* < 0.001 vs. scramble + H_2_O_2_ group. (c) Bands demonstrating the expression of SOD1 and SOD2 in each group. *β*-Actin was used as a loading control. (d) Semiquantitation of SOD1 from (c). *N* = 3, ^∗∗^*p* < 0.01 vs. scramble group; ^#^*p* < 0.05 vs. scramble + H_2_O_2_ group. (e) Semiquantification of SOD2 from (c). *N* = 3, ^∗∗^*p* < 0.01*vs*. scramble group; ^#^*p* < 0.05,^##^*p* < 0.01 vs. scramble + H_2_O_2_ group.

To further decipher the role of PTEN/Akt/mTOR signaling pathway in refreshing NSC proliferation potential that was suppressed by oxidative stress, shPTEN lentivirus was used to knock down the expression of PTEN in NSC. The expression of PTEN was markedly diminished with the application of shPTEN-1 and shPTEN-2 lentivirus (Figures 5(c) and 5(d)). Given that the higher efficiency of shPTEN-1 lentivirus, the shPTEN-1 lentivirus was utilized in our future experiments. Then, the effect of PTEN KD on oxidative stress was investigated. The results presented that, compared with scramble group, the fluorescence intensity of MitoROS in H_2_O_2_ group was obviously increased, while this effect was dramatically abolished with application of 5 *μ*M THF and/or shPTEN lentivirus (Figures 6(a) and 6(b)). Furthermore, the expression level of SOD1 and SOD2 in H_2_O_2_ group was obviously reduced, whereas this decreased expression level of SOD1 and SOD2 was abolished with the administration of 5 *μ*M THF and/or shPTEN lentivirus (Figures 6(c)–6(e)), to some extent. These results demonstrated that PTEN KD lentivirus exerted almost the same effect as 5 *μ*M THF in eliminating oxidative stress.

**Figure 7:**
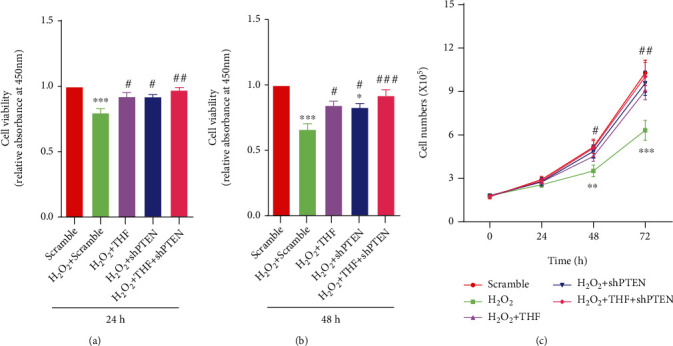
PTEN KD regained the inhibitory NSC proliferation capacity resulting from oxidative stress. (a) Bar graph summarizing the proliferation index of NSC using CCK8 at 24 h in each group. *N* = 6, ^∗∗∗^*p* < 0.001 vs. scramble group; ^#^*p* < 0.05, ^##^*p* < 0.01 vs. scramble + H_2_O_2_ group. (b) Histogram demonstrating the proliferation indice of NSC at 48 h using CCK8 in varying groups. *N* = 6, ^∗^*p* < 0.05, ^∗∗∗^*p* < 0.001 vs. scramble group; ^#^*p* < 0.05, ^###^*p* < 0.001 vs. scramble + H_2_O_2_ group. (c) Growth curve analysis in different groups. *N* = 5, ^∗∗^*p* < 0.01, ^∗∗∗^*p* < 0.001 vs. scramble group; ^#^*p* < 0.05, ^##^*p* < 0.01 vs. scramble + H_2_O_2_ group.

**Figure 8:**
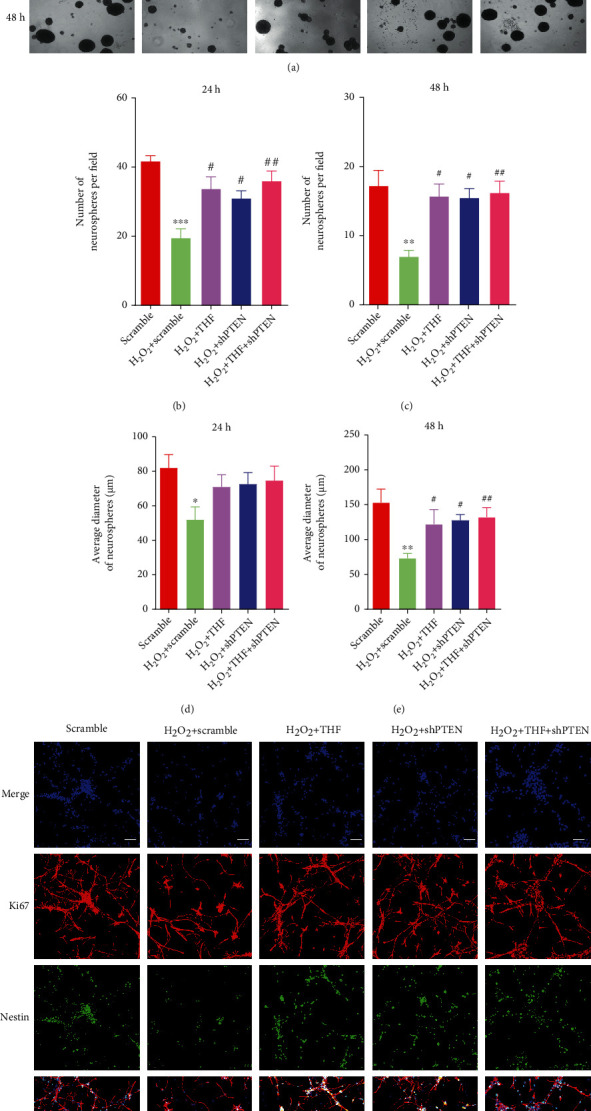
PTEN KD exerted the same effect as THF on retrieving NSC proliferation potential suppressed by oxidative stress. (a) The phase-contrasted images depicting NSC growth in each group at different timepoints. Scale bars: 50 *μ*m. (b) Bar graph summarizing the number of neurospheres per field among different groups at 24 h. *N* = 5, ^∗∗∗^*p* < 0.001 vs. scramble group; ^#^*p* < 0.05, ^##^*p* < 0.01 vs. scramble + H_2_O_2_ group. (c) Bar chart demonstrating the number of neurospheres per field among different groups at 48 h. *N* = 5, ^∗∗^*p* < 0.01 vs. scramble group; ^#^*p* < 0.05, ^##^*p* < 0.01 vs. scramble + H_2_O_2_ group. (d) Histogram illustrating the average diameter of neurospheres in each group at 24 h. *N* = 5, ^∗^*p* < 0.5 vs. scramble group. (e) Qualification of the average diameter of neurospheres in each group at 48 h. *N* = 5, ^∗∗^*p* < 0.01 vs. scramble group; ^#^*p* < 0.05, ^##^*p* < 0.01 vs. scramble + H_2_O_2_ group. (f) The immunostaining of Nestin and Ki67 in various groups. Scale bars: 50 *μ*m.

Afterward, the effect of PTEN KD on NSC proliferation was investigated. Firstly, the CCK8 assays demonstrated that the absorbance value in scramble, H_2_O_2_ + THF, H_2_O_2_ + shPTEN, and H_2_O_2_ + THF + shPTEN groups was higher than that in scramble + H_2_O_2_ group at 24 hours and 48 hours (Figures 7(a) and 7(b)). Meanwhile, the growth curve analysis indicated that PTEN KD restored proliferation ability of NSC subjected to oxidative stress, in a degree (Figure 7(c)). Further, neurosphere formation assays indicated that the number of neurospheres at 24 hours and 48 hours and the diameter at 48 hours in scramble, H_2_O_2_ + THF, H_2_O_2_ + shPTEN, and H_2_O_2_ + THF + shPTEN groups were profoundly increased compared with scramble + H_2_O_2_ group, while no significant difference was observed among scramble group, H_2_O_2_ + THF group, H_2_O_2_ + shPTEN group, and H_2_O_2_ + THF + shPTEN group (Figures 8(a)–8(e)). Additionally, the immunostaining of Nestin and Ki67 was applied to elucidate the role of THF in NSC proliferation initiated by oxidative stress. The immunostaining images depicted that the percentage of Nestin and Ki67 double-positive cells in H_2_O_2_ group was surely lower than that in scramble group, and the proportion of Nestin and Ki67 double-positive cells was significantly higher in H_2_O_2_ + THF, H_2_O_2_ + shPTEN, and H_2_O_2_ + THF + shPTEN groups, while no marked significance was observed among scramble group, H_2_O_2_ + THF group, H_2_O_2_ + shPTEN group, and H_2_O_2_ + THF + shPTEN group (Figure 8(f)). Collectively, these results demonstrated that administration of PTEN lentivirus alleviated the inhibitory effect on NSC expansion resulted from oxidative stress.

**Figure 9:**
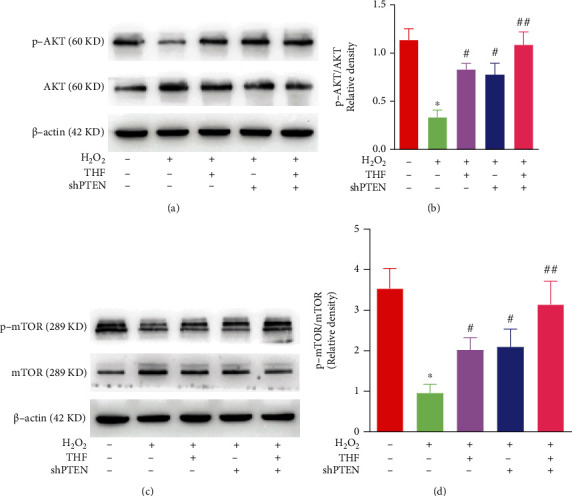
PTEN/Akt/mTOR signaling pathway played an evident role in THF mitigating the reduced NSC proliferation ability initiated by oxidative stress. (a) Immunoblot bands illustrating the expression of p-Akt and Akt in different groups. (b) Semiquantitative analysis of p-Akt/Akt from (a). *N* = 3, ^∗^*p* < 0.05, ^∗∗^*p* < 0.01 vs. scramble group; ^#^*p* < 0.05, ^##^*p* < 0.01 vs. scramble + H_2_O_2_ group. (c) Bands demonstrating the expression of p-mTOR and mTOR in each group. (d) Semiquantification of p-mTOR/mTOR from (c). *N* = 3, ^∗^*p* < 0.05, ^∗∗^*p* < 0.01 vs. scramble group; ^#^*p* < 0.05, ^##^*p* < 0.01 vs. scramble + H_2_O_2_ group.

Subsequently, the downstream effectors of Akt and mTOR were determined to test the role of PTEN/Akt/mTOR signaling pathway in regaining NSC proliferation potential attenuated by oxidative stress using Western blotting. The immunoblot bands illustrated that the reduced expression of p-Akt in H_2_O_2_ group was remarkably enhanced in H_2_O_2_ + THF, H_2_O_2_ + shPTEN, and H_2_O_2_ + THF + shPTEN groups, while no marked significance was observed among scramble group, H_2_O_2_ + THF group, H_2_O_2_ + shPTEN group, and H_2_O_2_ + THF + shPTEN group (Figures 9(a) and 9(b)). Meanwhile, the expression of p-mTOR demonstrated the same tendency as p-Akt among various groups (Figures 9(c) and 9(d)). Collectively, these results showcased that THF refreshed the restricted proliferating capacity of NSC under oxidative stress condition through PTEN/Akt/mTOR signaling pathway.

## 4. Discussion

In the present study, the results demonstrated that oxidative stress suppressed NSC proliferation, which was significantly alleviated by THF administration (5 *μ*M and 10 *μ*M). A negative relationship was found between PTEN expression and p-Akt or p-mTOR expression. Further evidence revealed that THF administration reduced the inhibitory effect of oxidative stress on NSC proliferation ascribing to activating the PTEN/Akt/mTOR signaling pathway.

The PTEN/Akt/mTOR signaling pathway is usually activated to exert multiple biological effects such as antioxidation when the ROS level is too high to beyond the physiologically antioxidative capability. Here, the results demonstrated that a high ROS level induced by 50 *μ*M H_2_O_2_ possessed the ability of increasing the expression of PTEN, while reducing the expression of p-Akt and p-mTOR, indicating that PTEN negatively mediates the Akt/mTOR signaling pathway, which is consistent with previous studies [39, 40]. Meanwhile, the results delineated that the expression of PTEN was upregulated when primary NSC were subjected to oxidative stress, which exists a discrepancy with previous studies [39, 40]. The reason for this phenomenon might be ascribed to the cell subtype difference as these researches focus on the terminally differentiated somatic cells such as mesothelial cells and retinal pigment epithelium (RPE) cells, instead of stem cells, which bear the potency of differentiating into terminally differentiated somatic cells [41].

NSC, one subpopulation of stem cells, maintain a homeostasis among self-renewal, differentiation, and quiescence under physiological condition [5], whereas NSC possess the capacity of proliferating *in situ*, migrating toward the injured regions, differentiating into three main neural subpopulations (neurons, astrocytes, and oligodendrocytes), and integrating into the damaged neurovascular network to initiate neuro-regenerative ability after CNS injury [6–9]. Our previous studies have identified some factors inhibiting the regenerative ability of NSC, such as actin alpha 2 (ACTA2) [8], G protein-coupled estrogen receptor 1 (GPER1) [2], and chondroitin sulfate proteoglycan (CSPG) [42]. Meanwhile, a few therapeutic strategies are developed to strengthen the rehabilitative potential of NSC, such as the ascorbic acid (AA) receptor-sodium-vitamin C cotransporter 2 (SVCT2), poly-L-ornithine (PO) [9, 38], ambroxol [7], repetitive transcranial magnetic stimulation (rTMS) [43], and 15d-PGJ2, an endogenous ligand of peroxisome proliferator-activated receptor *γ* (PPAR*γ*) [44]. In the present study, THF is proven to be an effective treatment recovering the inhibitory proliferation capacity of NSC resulting from oxidative stress.

Oxidative stress is a common pathology in varying CNS diseases. Previous studies have indicated that oxidative stress results in lipid peroxidation which leads to neural cells apoptosis in the cerebellum, hippocampus, and cerebral cortex after TBI [15, 16], cerebral stroke [17–19], and neurodegenerative diseases [20–22]. Herein, reducing oxidative stress is an effective approach to diminish loss of neural cells and enhance neuro-regenerative capacity. Here, the results certified that oxidative stress inhibited NSC proliferation to hinder the rehabilitative ability after CNS injury. Furthermore, THF has been validated to be a promising treatment in alleviating oxidative stress, therefore, recalling the inhibitory effect of oxidative stress on NSC proliferation.

THF, a crucial cofactor for the conversion of homocysteine to methionine, holds multifaced neuroprotective effects after CNS injury. Hyperhomocysteinemia, characterized as the elevated plasma levels of homocysteine, has been showed to be a risk factor for neurodevelopmental and neurodegenerative disorders. Research has demonstrated that THF application clearly reduces the increased level of homocysteine in plasma to decrease ROS production, thereafter, decreasing the apoptosis of neural cells [45]. Meantime, previous investigations have showcased that ROS accumulation initiates immune response which causes damage to neural cells after CNS injury [46, 47]. Furthermore, our previous report also shows that ROS accumulation triggers ferroptosis, and ferrostatin-1, a potent inhibitor of ferroptosis, inhibits ferroptosis in oligodendrocyte, finally reducing white matter injury and promoting functional recovery following spinal cord injury (SCI) in rats [48]. Additionally, investigation reveals that decreased THF lowers nucleotide biosynthesis and inhibits cell proliferation [49]. Hence, it is reasonable to believe that THF is a suitable candidate in treatment of CNS diseases as THF effectively reduces ROS cumulation that was validated in the current research.

## 5. Conclusions

In summary, the present study reveals that THF administration prominently abrogates the inhibitory effect of oxidative stress on NSC proliferation via diminishing ROS level in mouse primary NSC. And the underlying mechanism favoring of this phenomenon is that oxidative stress activates the PTEN/Akt/mTOR pathway, and PTEN serves as a negative mediator decreasing the expression of p-Akt expression and its downstream effector of p-mTOR. The present study presents a rationale for administrating THF to potentiate the rehabilitative capacity of NSC in the treatment of CNS diseases with the presence of oxidative stress.

## Data Availability

The data used to support the findings of this study are available from the corresponding author upon reasonable request.
